# Acute Necrotizing Pancreatitis Secondary to Adhesive Afferent Loop Syndrome Six Years After Total Gastrectomy With Roux-en-Y Reconstruction: A Case Report

**DOI:** 10.7759/cureus.113594

**Published:** 2026-07-29

**Authors:** Emilly Hanay M Santos, Ana Carolina B Blitzkow

**Affiliations:** 1 General Surgery and Digestive Surgery, Hospital de Clínicas Universidade Federal do Paraná, Curitiba, BRA

**Keywords:** acute pancreatitis, adhesions, afferent loop syndrome, gastrectomy, roux-en-y reconstruction

## Abstract

Acute pancreatitis is an uncommon presentation of afferent loop syndrome, and progression to necrotizing pancreatitis is rarely described in the literature. A 65-year-old man presented with severe epigastric pain, nausea, and bilious vomiting six years after total gastrectomy with Roux-en-Y reconstruction for gastric adenocarcinoma. Laboratory tests showed marked elevations of amylase and lipase. Initial contrast-enhanced computed tomography (CT) demonstrated afferent loop obstruction with focal pancreatic hypoenhancement. Repeat CT performed approximately 72 hours after symptom onset confirmed progression to necrotizing pancreatitis. Emergency exploratory laparotomy identified an adhesive band causing mechanical obstruction of the afferent limb, and adhesiolysis successfully restored intestinal transit. The patient recovered uneventfully, with progressive clinical and laboratory improvement, and remained asymptomatic at the six-month follow-up. This case highlights that afferent loop syndrome should be considered in patients with previous gastric reconstruction who present with acute pancreatitis, even years after surgery. Early contrast-enhanced CT, together with prompt surgical intervention when indicated, is essential for identifying the underlying mechanical obstruction and improving patient outcomes.

## Introduction

Afferent loop syndrome was first described in 1950 and is an uncommon complication caused by mechanical obstruction of the afferent loop after Billroth II gastrectomy [1] or of the biliopancreatic (afferent) limb after Roux-en-Y reconstruction, in which the afferent limb carries bile and pancreatic secretions while the alimentary (efferent) limb transports ingested food [2,3], with a reported incidence of 0.2% to 1% depending on the type of reconstruction performed [2-4]. Despite its low incidence, afferent loop syndrome requires prompt recognition because delayed diagnosis and treatment are associated with high mortality rates [2].

Mechanical obstruction of the afferent loop has several possible causes, including intraluminal, intramural, or extrinsic causes, such as enteroliths, bezoars, ulcer-related fibrosis, adhesions, internal hernias, volvulus, intussusception, and tumor recurrence [2-4]. Patients typically present with epigastric pain, nausea, and bilious vomiting, although the clinical presentation may closely resemble acute pancreatitis, making diagnosis particularly challenging [2].

Although afferent loop syndrome is a recognized cause of acute pancreatitis, progression to necrotizing pancreatitis appears to be rarely reported, particularly in delayed presentations years after Roux-en-Y reconstruction. This report describes a patient who developed acute necrotizing pancreatitis secondary to adhesive afferent loop syndrome six years after total gastrectomy for gastric adenocarcinoma. Contrast-enhanced computed tomography (CT) was crucial in demonstrating pancreatic necrosis and identifying the underlying adhesive mechanical obstruction, which was confirmed intraoperatively. Prompt surgical treatment led to complete recovery, highlighting the importance of early recognition and intervention in this uncommon but potentially life-threatening complication.

## Case presentation

A 65-year-old man with a history of total gastrectomy with Roux-en-Y reconstruction for gastric adenocarcinoma six years earlier and previous cholecystectomy presented to the emergency department with a one-day history of severe epigastric pain, cessation of bowel movements, nausea, and bilious vomiting that worsened after meals. He had no other known comorbidities, denied alcohol consumption and smoking, had no known drug allergies, and was not taking regular medications.

On admission, the patient was afebrile, with a blood pressure of 135/76 mmHg, heart rate of 83 beats/min, respiratory rate of 20 breaths/min, oxygen saturation of 92% on room air, and a pain score of 8/10. Physical examination revealed diffuse abdominal tenderness without palpable masses or signs of peritonitis. Blood samples were obtained on admission, and the initial laboratory findings are summarized in Table 1. The most significant abnormalities were leukocytosis (15,440/mm³ with 20% band forms), elevated pancreatic enzymes (amylase 8,003 U/L and lipase 6,000 U/L), and increased liver transaminases.

**Table 1:** Laboratory findings at admission and during postoperative follow-up. Laboratory results obtained at hospital admission and during postoperative follow-up. Bold values indicate abnormal laboratory findings according to the reference range. Serial measurements demonstrate progressive biochemical improvement following surgical relief of the afferent loop obstruction.

Laboratory parameter	Reference range	Admission	Postoperative*
Hemoglobin (g/dL)	13.5–17.5	15.3	12.4
Hematocrit (%)	41–53	45.0	37.1
Leukocyte count (/mm³)	4,000–11,000	15,440	11,220
Band neutrophils (%)	0–5	20	21
Platelet count (/mm³)	150,000–450,000	187,000	176,000
Creatinine (mg/dL)	0.72–1.3	0.97	0.87
Urea (mg/dL)	15–45	43	44
Sodium (mmol/L)	136–145	139	140
Potassium (mmol/L)	3.5–5.1	4.8	3.8
Total bilirubin (mg/dL)	0.2–1.2	1.38	0.26
Direct bilirubin (mg/dL)	0.0–0.3	0.83	0.28
Aspartate transferase (U/L)	<40	847	—
Alanine transaminase (U/L)	<41	651	—
Albumin (g/dL)	3.5–5.0	3.9	—
International normalized ratio	0.8–1.2	1.26	—
Amylase (U/L)	25–125	8,003	371
Lipase (U/L)	≤60	6,000	67

**Figure 1:**
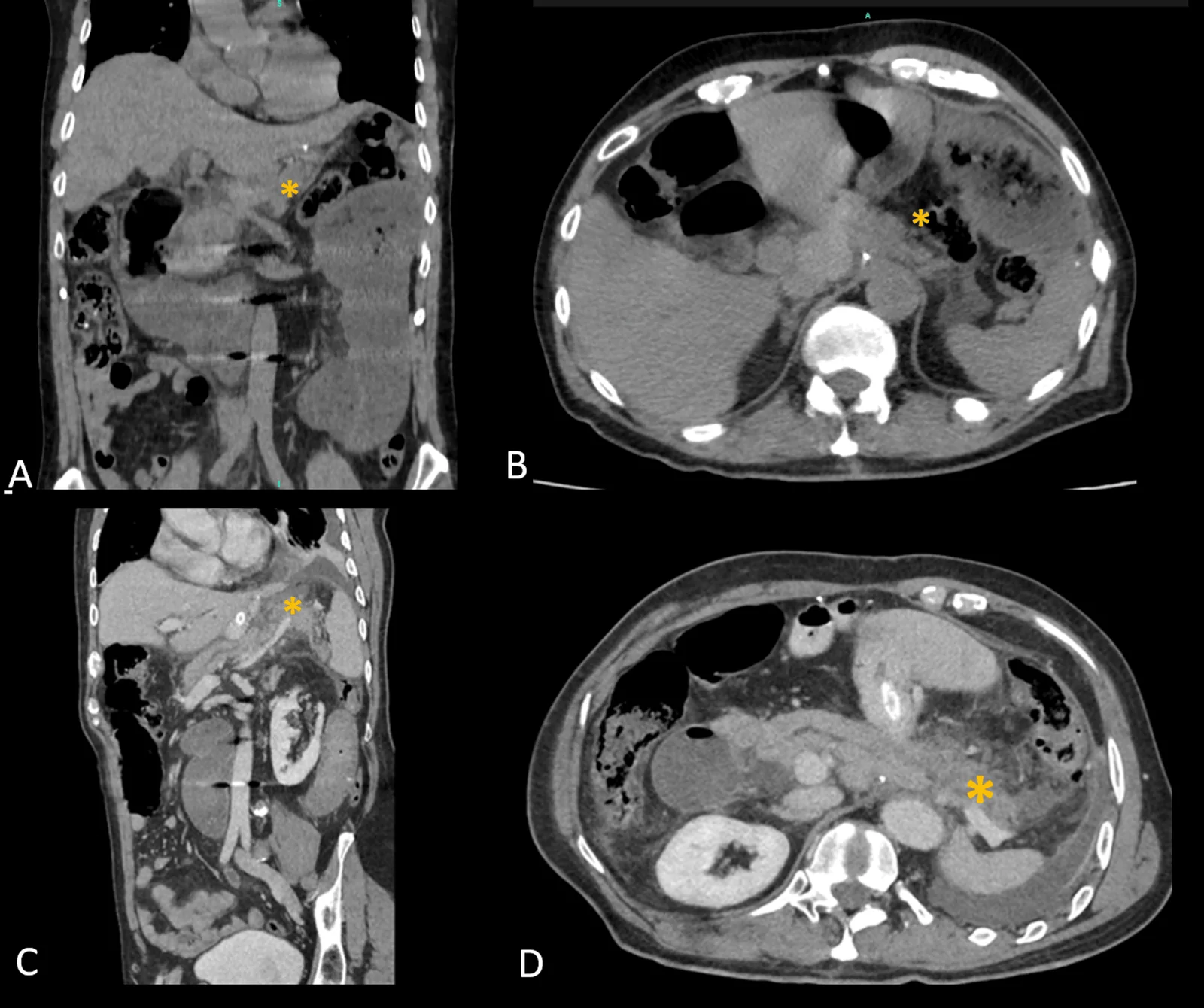
Contrast-enhanced abdominal CT demonstrating the evolution of pancreatic findings. A and B: Abdominal CT obtained approximately 24 hours after symptom onset showing focal pancreatic hypoenhancement (yellow asterisk). C and D: Repeat CTs were obtained approximately 72 hours after symptom onset, showing progression to extensive non-enhancing pancreatic parenchyma (yellow asterisk), consistent with necrotizing pancreatitis. Coronal (A), coronal oblique (C), and axial (B and D) images are shown.

A contrast-enhanced abdominal CT performed approximately 24 hours after symptom onset demonstrated marked dilatation of the duodenum (up to 43 mm) and the afferent jejunal limb (up to 75 mm) with fecalized content and an abrupt transition point adjacent to the anterior abdominal wall, suggesting afferent loop obstruction. The pancreatic body-tail junction showed a focal 25-mm area of hypoenhancement without pancreatic duct dilatation, interpreted as a focal perfusion abnormality of uncertain significance (Figure 1).

At initial presentation, the patient showed no evidence of bowel ischemia, perforation, hemodynamic instability, or generalized peritonitis that would require immediate surgical intervention. Therefore, conservative management was initiated with bowel rest (nil per os), intravenous fluid resuscitation, analgesia, and nasogastric tube decompression.

**Figure 2:**
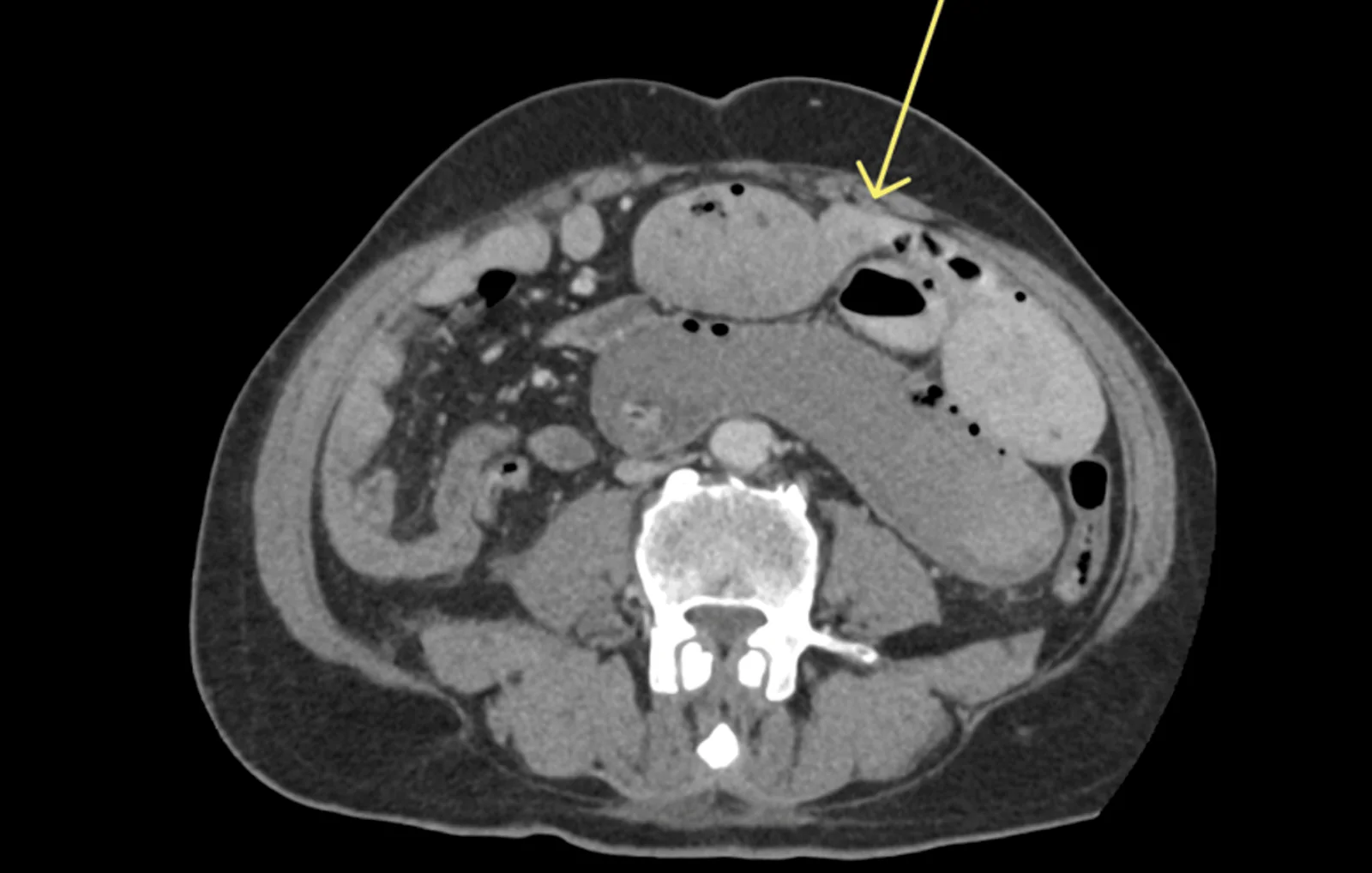
Axial contrast-enhanced abdominal CT performed approximately 72 hours after symptom onset demonstrating the abrupt caliber transition of the jejunal loop adjacent to the anterior abdominal wall (yellow arrow), corresponding to the site of mechanical obstruction.

**Figure 3:**
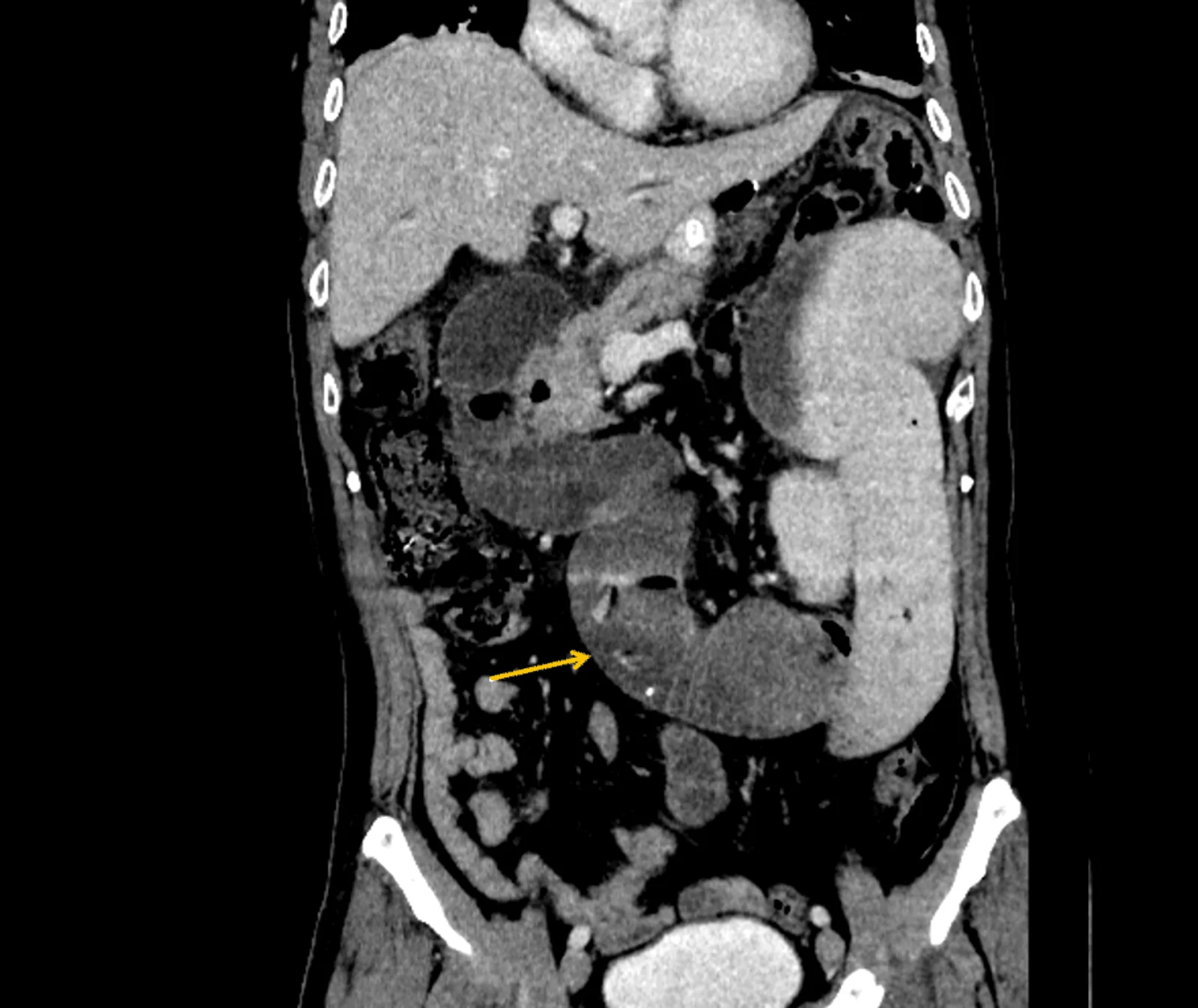
Coronal contrast-enhanced CT performed approximately 72 hours after symptom onset demonstrating dilatation of the duodenum and afferent jejunal limb (yellow arrow). Distal ileal loops are collapsed, while orally administered contrast reaches only the proximal ileum.

Despite these measures, the patient's abdominal pain progressively worsened. Given the persistent clinical deterioration, repeat contrast-enhanced CT was performed approximately 72 hours after symptom onset. The examination demonstrated progression to necrotizing pancreatitis, with non-enhancing pancreatic parenchyma involving more than 30% of the pancreatic body and tail (approximately 83 mm), accompanied by new peripancreatic inflammatory changes (Figure 1). The modified CT Severity Index (MCTSI) was 8/10, consistent with severe acute pancreatitis [5]. Persistent dilatation of the duodenum (up to 43 mm) and the afferent jejunal limb (up to 75 mm), together with a well-defined transition point on serial CT examinations, favored a mechanical obstruction rather than functional bowel dilatation, prompting emergency exploratory laparotomy (Figures 2, 3).

Given the persistent absence of bowel movements, worsening abdominal pain, and progression to necrotizing pancreatitis despite conservative management, emergency exploratory laparotomy was performed. Intraoperatively, the afferent jejunal limb was markedly distended but remained viable, with no evidence of bowel ischemia or perforation. An adhesive band extending between the afferent jejunal limb and the anterior abdominal wall, located approximately 20 cm distal to the jejunojejunal anastomosis, was identified as the cause of the mechanical obstruction, confirming adhesive afferent loop syndrome. Adhesiolysis restored intestinal transit, and bowel resection was not required. Following surgery, the patient was admitted to the intensive care unit, where he was extubated and remained hemodynamically stable without vasoactive support.

The postoperative course was uneventful. After one day in the intensive care unit, the patient was transferred to the surgical ward. A liquid diet was initiated on postoperative day (POD) 1 and gradually advanced as tolerated. Abdominal pain resolved rapidly after surgery, and bowel function returned on POD 4.

Serial laboratory tests demonstrated progressive improvement paralleling the clinical recovery. Serum amylase decreased from 8,003 U/L at admission to 371 U/L on POD 9, while lipase declined from 6,000 U/L to 67 U/L (Table 1). These findings were consistent with resolution of pancreatitis following relief of the mechanical obstruction.

The patient was discharged on POD 9. At the six-month outpatient follow-up, he remained asymptomatic, with no recurrent abdominal pain, vomiting, or episodes of pancreatitis. Routine oncologic surveillance also showed no evidence of recurrent gastric cancer.

## Discussion

In patients with afferent loop obstruction, impaired drainage of biliary and pancreatic secretions results in progressive accumulation of fluid within the afferent limb and increased intraductal pressure. Persistent ductal hypertension promotes premature intrapancreatic activation of trypsinogen, initiating a cascade of digestive enzyme activation that leads to acinar cell injury. The resulting inflammatory response contributes to microcirculatory dysfunction, tissue ischemia, and increased vascular permeability, which may ultimately progress to pancreatic necrosis in severe cases [4,6].

Diagnosing afferent loop syndrome can be challenging because its clinical presentation may overlap with that of acute pancreatitis [6-10]. In the present case, the patient presented with severe epigastric pain, bilious vomiting, markedly elevated pancreatic enzyme levels, and CT evidence of afferent limb dilatation. Nevertheless, the absence of bowel ischemia, perforation, hemodynamic instability, or generalized peritonitis supported an initial conservative approach. Continued clinical deterioration prompted repeat contrast-enhanced CT, which demonstrated progression to necrotizing pancreatitis, persistent dilatation of the afferent jejunal limb, and a well-defined transition point. Unlike functional bowel dilatation, which typically presents as diffuse bowel distension without a discrete transition point, mechanical afferent loop obstruction is characterized by localized dilatation of the afferent limb with an identifiable site of obstruction [3]. By simultaneously demonstrating these features and the associated pancreatic complications, CT raised suspicion for afferent loop syndrome and guided timely surgical exploration. Exploratory laparotomy subsequently identified an adhesive band causing mechanical obstruction of the afferent jejunal limb, without evidence of bowel ischemia or perforation, thereby confirming the diagnosis and allowing definitive treatment by adhesiolysis.

Surgical intervention has traditionally been considered the mainstay of treatment for acute afferent loop syndrome. However, the choice between laparotomy and laparoscopy should be individualized according to the patient's clinical condition, imaging findings, and the surgeon's expertise [11,12]. In the present case, emergency exploratory laparotomy was selected because of the patient's progressive clinical deterioration, development of necrotizing pancreatitis, and the need for definitive evaluation of bowel viability and prompt relief of the mechanical obstruction.

Although afferent loop syndrome is an uncommon complication after gastric reconstruction, its reported incidence after Roux-en-Y reconstruction is approximately 0.2% [10]. Acute presentations are classically observed in the early postoperative period, although delayed cases may occur and should not be overlooked. Most reported cases develop within the first months after surgery [7,10,13], although rare cases have been described as late as 15 years after surgery [13]. In the present case, afferent loop syndrome developed six years after total gastrectomy, emphasizing that this diagnosis should remain in the differential diagnosis regardless of the time elapsed since gastric reconstruction.

Few cases of acute pancreatitis secondary to adhesive afferent loop syndrome have been reported in the literature [12-18]. Delayed presentation associated with Roux-en-Y reconstruction and pancreatic necrosis appears to be particularly uncommon. Reported management strategies varied according to disease severity and the patient's clinical condition. Most patients underwent exploratory laparotomy [13-17], although laparoscopic management has also been described [12]. Conservative treatment with intravenous fluid resuscitation and nasogastric decompression has been reported in selected patients [14].

Because delayed diagnosis and treatment are associated with mortality rates ranging from 30% to 60% [2], afferent loop syndrome should be considered in the differential diagnosis of acute pancreatitis in patients with a history of gastric reconstruction, particularly when imaging suggests mechanical obstruction of the afferent loop.

## Conclusions

Afferent loop syndrome is a rare but important cause of acute pancreatitis that should be considered in the differential diagnosis of patients with previous gastric reconstruction, including Roux-en-Y reconstruction, who present with symptoms of acute pancreatitis. As illustrated by this case, the condition may be mistaken for primary acute pancreatitis because of its overlapping clinical presentation. Careful interpretation of contrast-enhanced CT in conjunction with the patient's surgical history and intraoperative findings was essential to identify the underlying adhesive mechanical obstruction and guide definitive management, resulting in progressive clinical and biochemical recovery.
